# The Landscape of Candidate Driver Genes Differs between Male and Female Breast Cancer

**DOI:** 10.1371/journal.pone.0078299

**Published:** 2013-10-23

**Authors:** Ida Johansson, Markus Ringnér, Ingrid Hedenfalk

**Affiliations:** Division of Oncology, Department of Clinical Sciences, Lund and CREATE Health Strategic Center for Translational Cancer Research, Lund University, Lund, Sweden; Florida International University, United States of America

## Abstract

The rapidly growing collection of diverse genome-scale data from multiple tumor types sheds light on various aspects of the underlying tumor biology. With the objective to identify genes of importance for breast tumorigenesis in men and to enable comparisons with genes important for breast cancer development in women, we applied the computational framework COpy Number and EXpression In Cancer (CONEXIC) to detect candidate driver genes among all altered passenger genes. Unique to this approach is that each driver gene is associated with several gene modules that are believed to be altered by the driver. Thirty candidate drivers were found in the male breast cancers and 67 in the female breast cancers. We identified many known drivers of breast cancer and other types of cancer, in the female dataset (e.g. *GATA3*, *CCNE1*, *GRB7*, *CDK4*). In contrast, only three known cancer genes were found among male breast cancers; *MAP2K4*, *LHP*, and *ZNF217*. Many of the candidate drivers identified are known to be involved in processes associated with tumorigenesis, including proliferation, invasion and differentiation. One of the modules identified in male breast cancer was regulated by *THY1*, a gene involved in invasion and related to epithelial-mesenchymal transition. Furthermore, men with *THY1* positive breast cancers had significantly inferior survival. *THY1* may thus be a promising novel prognostic marker for male breast cancer. Another module identified among male breast cancers, regulated by *SPAG5*, was closely associated with proliferation. Our data indicate that male and female breast cancers display highly different landscapes of candidate driver genes, as only a few genes were found in common between the two. Consequently, the pathobiology of male breast cancer may differ from that of female breast cancer and can be associated with differences in prognosis; men diagnosed with breast cancer may consequently require different management and treatment strategies than women.

## Introduction

Male breast cancer (MBC) is a rare cancer form that has not been well studied, and there is hence limited knowledge of the disease at the genetic and molecular levels. A few molecular profiling studies of MBC have been performed by us and others [1-4], but the rarity of the disease makes the collection of large sample cohorts difficult. Although MBC shares many similarities with female breast cancer (FBC), distinct differences have been reported in age distribution, levels of hormone receptors, prognosis and survival [5-7], and on the transcriptional and genomic levels [1-4]. No large comprehensive studies of the etiology of the disease or randomized trials for optimizing patient management have been conducted to date, and MBC is often likened to hormone receptor-positive disease in postmenopausal women [8]. Thus, recommendations for managing MBC have been based on available knowledge and guidelines for FBC. Several studies have nevertheless reported various differences between MBC and FBC based on gene expression (GEX) [2,4], copy number (using array comparative genomic hybridization, aCGH) [1,3], microRNA [9,10] and SNP levels [11]. Among these, we have shown in two previous studies that MBC, like FBC, is a heterogeneous disease, and many differences between MBC and FBC were revealed [1,2]. In a transcriptional profiling study we described two new subgroups of breast cancer (Luminal M1 and M2, respectively) that did not resemble any of the intrinsic subgroups reported in FBC, and as such may be unique to males. Luminal M1 tumors (70% of the MBC tumors) seemed to be more aggressive and were associated with worse prognosis and also appeared to have a less activated estrogen receptor (ER) module, while Luminal M2 tumors (30% of the MBC tumors) displayed an up-regulated immune response and a more activated ER module [2]. These findings suggest that MBCs are in fact different from FBCs, and that men diagnosed with breast cancer may require other treatment strategies than women. 

Many methods are currently available for producing various types of genome-scale data, and the amount of such data from human cancers is growing rapidly. There is however a need to combine the different types of data, reflecting various aspects of tumor biology, to gain a better and deeper understanding of the underlying biology [12]. In this context, one major challenge is to combine copy number and transcriptional data in a biologically meaningful fashion. Various methods for integrating copy number and transcriptional data are available, all aimed at differentiating between driver and passenger genes [13-15]. In this study, we used the computational framework COpy Number and EXpression In Cancer (CONEXIC), which integrates aCGH and GEX data to identify cancer drivers among all passenger genes aberrantly expressed in tumors. This approach not only identifies candidate drivers, but also associates each driver with gene modules that are believed to be altered by the driver [13]. In contrast, most previously described network-based methods have focused only on a single type of genome-scale data [16,17].

The main aim of this study was to identify potential candidate driver genes that drive proliferation and metastasis, thereby distinguishing them from passenger genes, in MBC, an approach that has not been comprehensively explored before. Secondly, we aimed to compare the landscape of potential candidate drivers between breast cancers diagnosed in male and female patients to explore differences as well as similarities. 

## Materials and Methods

### Ethics statement

The study was approved by the regional Ethics Committee in Lund (2012/89), waiving the requirement for informed consent for the present study.

### Patient material

The fresh frozen MBC tissues were obtained from The Southern Sweden Breast Cancer Group’s tissue bank at the Department of Oncology, Skåne University Hospital, from Uppsala University Hospital, and Örebro Hospital, all in Sweden. Patients were diagnosed between 1983 and 2009. All cases with good quality aCGH [1] and GEX [2] data from our previous studies were included in the present study (n=53). A physician reviewed all patient charts and compiled all clinico-pathological data. A pathologist graded all tumors to current pathological standard when paraffin blocks were available; all histological grades were represented. ER, progesterone receptor (PR) and HER2 were re-evaluated (see 7,18 for further details). The patients had received different combinations of adjuvant treatment, including hormonal, chemotherapy and radiation treatment (see Table 1 for further information).

**Table 1:** Patient and tumor characteristics of the 53 MBC cases.

**Clinico-pathological characteristics**		**N**	**%**
Age at diagnosis	Mean	68	
	Range	42-92	
ER status	Positive	41	77
	Negative	3	6
	N/A	9	17
PR status	Positive	36	68
	Negative	8	15
	N/A	9	17
*HER2* status	Positive	2	4
	Negative	27	51
	N/A	24	45
*BRCA2* mutation status	Positive	3	6
	Negative	6	11
	N/A	44	83
Histology	DCIS	1	2
	Invasive cancer in combination with DCIS	9	17
	Invasive cancer	38	72
	N/A	5	9
Histological grade	I	2	4
	II	22	42
	III	11	21
	N/A	18	34

The FBC cohort included 359 tumors, representing all intrinsic subgroups of FBC and is described in [19].

### Bioinformatic analyses

The computational framework CONEXIC, that integrates aCGH and GEX data for detecting candidate drivers, was used. CONEXIC uses an integrative Bayesian scoring approach [13] and is inspired by Module Networks [20]. The basic assumptions for CONEXIC are:

1 Drivers should modulate the gene modules’ expression.
2 The driver’s expression controls the malignant phenotype rather than the copy number, since also other mechanisms can cause altered expression of the driver.

For a detailed description of the CONEXIC method, see Akavia et.al. [13]. Briefly, the learning algorithm of CONEXIC consists of three main steps:

1 Selection of candidate drivers from commonly aberrant regions of the genome.
2 A Single Modulator step that finds the initial association between the candidate drivers and gene modules.
3 Improvement of the Single Modulator modules by an iterative network learning step.

The 53 MBCs and 359 FBCs with paired GEX and aCGH data were analyzed in the same manner. First, JISTIC (identification of significant targets of cancer) was used to identify commonly aberrant regions (i.e. more often than what would be expected by chance) of the genome in the tumors [21]. JISTIC is an improvement of the GISTIC (Genomic Identification of Significant Targets In Cancer) algorithm [22], and uses both the frequency and magnitude of the alterations in the genome to calculate a statistical score. GISTIC has problems in identifying sub-regions when large regions are amplified or deleted, while JISTIC includes an algorithm with the capability to find these regions [21].

**Figure 1:**
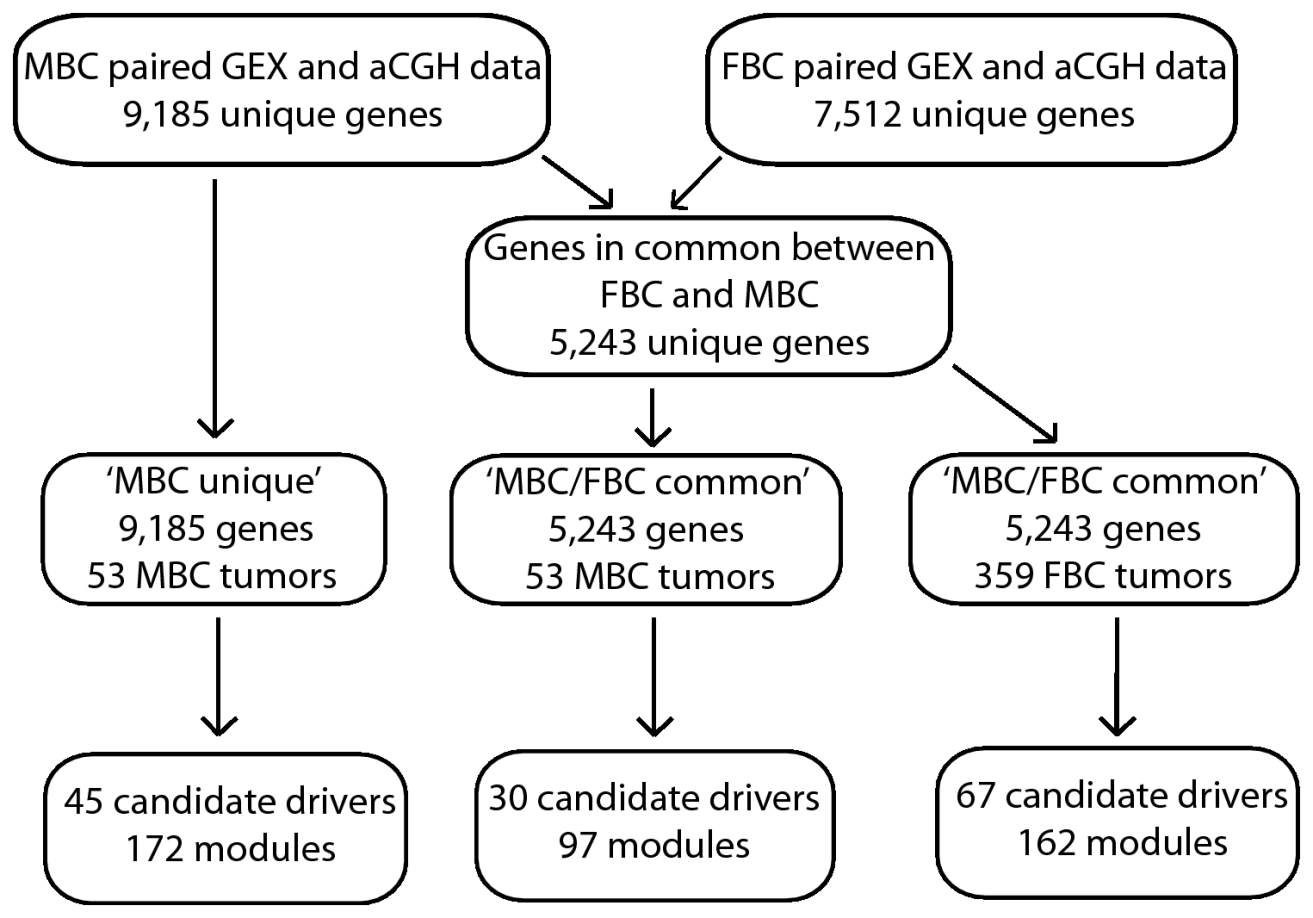
Flowchart outlining the steps in the CONEXIC analysis.

The GEX datasets were normalized as described before [2,19]. Probes with a standard deviation <0.25 on a log_2_ scale were removed and probe sets for genes were merged (inconsistent genes were removed). Genes without aCGH data were filtered out, resulting in a gene set of 9,185 unique genes for the MBCs and a gene set of 7,512 unique genes for the FBCs. Genes in common between the MBC and FBC datasets were then selected, resulting in a final set of 5,243 genes. CONEXIC was applied to the MBCs based on both the ‘MBC/FBC common’ (5,243 genes) and the ‘MBC unique’ (9,185 genes) gene sets (Figure 1).

The candidate driver lists were used in the Single Modulator step to establish an initial association to gene modules; non-parametric bootstrapping was used [23] and repeated 100 times. The candidate drivers that were selected in at least 90% of the runs were then used as candidate gene lists for the final run of the Single Modulator step. The result file was then split into two modules per candidate driver, one module each for genes positively and negatively correlated with the candidate driver. Genes that were not assigned to any gene module were clustered using K-means; 20 stable K-means clusters were found in the MBC dataset and five in the FBC dataset. The final modules from Single Modulator (after the split) and the K-means modules were used as a starting point in the Module Network step, and then by using an iterative approach each gene was re-assigned into a module that best modeled its behavior. The Bayesian score is improved at each iteration and also here a non-parametric bootstrap was used and repeated 100 times. Candidate drivers that were selected in at least 40% of the runs were used as the candidate gene lists for the final run of the Module Network step. To rank the candidate drivers from the final list, CONEXIC scores were calculated. The candidate driver with the highest CONEXIC score is the most probable driver. The CONEXIC score was calculated with a Normal Gamma score; a score is calculated for each module and the scores for all modules regulated by a candidate driver were summed up to generate the CONEXIC score.

Each driver is associated with gene modules and a threshold expression level (split value) of the driver for each gene module divides the tumors into two groups, one with tumors that express the driver below the threshold and one with tumors that express the driver above the threshold. 

An automated procedure, Literature Vector Analysis (LitVAn), was used to find over-represented terms associated with the genes in the modules in scientific papers from the NCBI Gene database [13].

CONEXIC was also applied separately to FBCs in the respective intrinsic subgroups (101 Luminal A, 65 Luminal B, 78 Basal-like and 42 HER2-enriched, respectively) and it was run in the same manner as described above. Unfortunately, the limited sample size precluded this possibility in the two previously identified subgroups of MBC (Luminal M1 and M2) [2].

Finally, a ranked-based module activity score was calculated for each MBC tumor for eight gene modules reflecting biologically relevant transcriptional programs found in FBC as previously described [24]. 

The GEX and aCGH data have been deposited in NCBI’s Gene Expression Omnibus (GEO) and are available through GEO Series accession numbers [GSE31259] and [GSE50512], respectively. 

### Statistical analyses

All statistical calculations and figures were generated in R [25]. The survival and survcomp packages were used for the survival with distant metastasis free survival (DMFS) as end-point. All P-values are two-sided. 

## Results

### JISTIC results

A JISTIC analysis was performed to find significantly aberrant regions among the MBC tumors, whereby 67 significant genomic aberrations were identified (51 gains and 16 losses). The regions contain 1,223 genes (769 gained and 454 lost). By comparison, when GISTIC was applied to the same tumors, only 39 regions (25 gains and 14 losses) were found [1]. Correspondingly, 208 significant regions of genomic aberrations were found (89 gained and 119 lost), harboring 924 genes (450 gained and 474 lost) when JISTIC was applied to the FBC tumors.

### CONEXIC results in MBC and FBC

Thirty candidate drivers were found among the MBC tumors using the ‘MBC/FBC common’ gene set (Table 2 and Table S1) and 67 among the FBC tumors (Table 3 and Table S2). The 30 candidate drivers identified in the MBC tumors regulated 97 modules and the 67 candidate drivers identified in the FBC tumors regulated 162 modules (Figure 1). Male and female breast cancers displayed remarkably different landscapes of candidate drivers, as only two candidate drivers were found in common (*TAF4* and *CD164*). When CONEXIC was applied separately to the intrinsic subgroups of FBC, only three more candidate drivers in common with MBC were found (*ARHGAP30*, *COG3* and *SPAG5*). This is in line with our previous results on transcriptional and copy number profiling, where we showed that MBCs appeared to differ from the intrinsic subgroups of FBC, and furthermore that MBCs could be divided into novel, distinct subgroups [1,2].

**Table 2:** Top 15 candidate driver genes in male breast cancer.

Candidate drivers MBC*	**CONEXIC score**	**Cytoband**	LitVAn**
**Amplified**			
BLCAP	2,544	20q11.23	Hypoxia, vascular, invasion, mitosis, cyclin, notch
LAD1	1,209	1q32.1	Vascular, hypoxia, notch, macrophages
CYC1	1,202	8q24.3	Vascular, notch
DDX51	1,180	12q24.33	Invasive, angiogenesis, collagen
ARHGAP30	1,085	1q23.3	Invasive, collagen, tnf, MHC
SPAG5	823	17q11.2	Mitosis, cyclin
TAF4	693	3p14.1	Cyclin, p53
**Deleted**			
ELAC2	1,960	17p11.2	Vesicle
THY1	1,586	11q23.3	Invasion, angiogenesis, collagen, mmp, integrin
LHFP	1,309	13q12	Mitosis, cyclin, mitosis, p53, tnf, MHC
CD164	1,016	6q21	Collagen
POSTN	815	13q13.3	Invasive, angiogenesis, vascular, collagen
ELF1	802	13q13	Hypoxia
FYN	715	6q21	Macrophage, tnf, MHC, collagen
LAMA4	607	6q21	Invasion, vascular, collagen

* Blue represents genes in regions with genomic losses and red represents genes in regions with genomic gains. ** LitVAn, Literature Vector Analysis.

**Table 3:** Top 15 candidate driver genes in female breast cancer.

Candidate drivers FBC*	**CONEXIC score**	**Cytoband**	LitVAn**
**Amplified**			
GATA3	15,464	10p15	Invasion, mcf, integrin
TIMP2	10,917	17q25	Invasion, angiogenesis, metastasis, collagen
APOM	9,563	6p21.33	TNF, p53
POLR2F	9,346	22q13.1	Lysine
NCAPG2	8,994	7q36.3	Invasion, cyclin, checkpoint, notch, metastasis, collagen, p53
CD4	8,299	12p13.31	Invasion, MHC, notch
AIF1	7,723	6p21.3	Invasion
KIFC1	6,012	6p21.3	Mitochondrial
PRR7	5,767	5q35.3	Cyclin, p53,
CSNK2B	5,107	6p.21.3	p53
**Deleted**			
ARHGAP19	15,179	10q24.1	Macrophage, MHC, cyclin, checkpoint, TNF
YIF1B	10,200	19q13.2	Invasion, collagen, notch, p53, vascular
DIAPH3	8,176	13q21.2	Invasion, collagen, cyclin, checkpoint, p53
TCF4	5,901	18q21.1	Invasion, vascular, MHC, TNF
NISCH	4,932	3p21.1	Invasion, collagen, vascular

* Blue represents genes in regions with genomic losses and red represents genes regions with genomic gains. ** LitVAn, Literature Vector Analysis.

When CONEXIC was applied to MBCs using the ‘MBC unique’ gene set, 45 candidate drivers were identified (Figure 1) and 17 of these were shared with the 30 candidate drivers from the ‘MBC/FBC common’ gene set (17/30 (57%)). Reassuringly, among the top 15 candidate drivers from the ‘MBC unique’ gene set, 11/15 (73%) were also found in the ‘MBC/FBC common’ gene set (data not shown).

CONEXIC analyses of the intrinsic subgroups of FBC identified 28 candidate drivers in Luminal A tumors, 17 in Luminal B tumors, 22 in Basal-like tumors and 12 in HER2-enriched tumors (data not shown). Many of these candidate drivers were also detected when all FBC tumors were analyzed together. As an example, *GATA3* was found as the top candidate driver among both Luminal A and Luminal B tumors. The landscapes of candidate drivers differed greatly between the intrinsic subgroups of FBC, and only *ARHGAP30* (Basal-like and HER2), *TAF4* (Basal-like), *SPAG5* (HER2 and Luminal A) and *COG3* (Luminal A) were shared with MBC.

### CONEXIC identifies known drivers of FBC

In order to highlight known oncogenes and tumor suppressor genes in the candidate gene lists, these were compared with the cancer Gene Census list (COSMIC, Catalogue of Somatic Mutations In Cancer) and the Amplified and Overexpressed Genes In Cancer (AOGIC) list. COSMIC to date contains 487 genes and AOGIC contains 77 genes. Eight known cancer genes from these compiled gene lists were found among the FBC candidate drivers: *GATA3*, *CCNE1*, *PRCC*, *MALM2*, *CDK4*, *GRB7*, *NFIB* and *VTI1A*. Among these, *GATA3*, *CCNE1*, *CDK4* and *GRB7* are known drivers of breast cancer [26,27]. In contrast, only two known cancer genes, *ZNF217* and *LHFP*, were identified among the thirty MBC candidate driver genes. When CONEXIC was applied to MBCs using the extended ‘MBC unique’ gene set, an overall similar landscape of candidate drivers for MBC was detected. In addition, *MAP2K4*, which was not included in the ‘MBC/FBC common’ gene set, was detected as a candidate driver in MBC (data not shown). *MAP2K4* has previously been reported to be over-expressed in pancreatic, breast and colorectal cancer, while *LHFP* expression has been reported in lipomas [26]. Interestingly, *GATA3* was not found to be a candidate driver for MBC in the present study, despite the fact that the majority of the MBC tumors were ER positive. *GATA3* was however identified as a candidate driver in the analyses of all FBCs, and within the Luminal A and Luminal B subtypes of FBC. 

### Biological processes and pathways regulated by the candidate drivers

**Figure 2:**
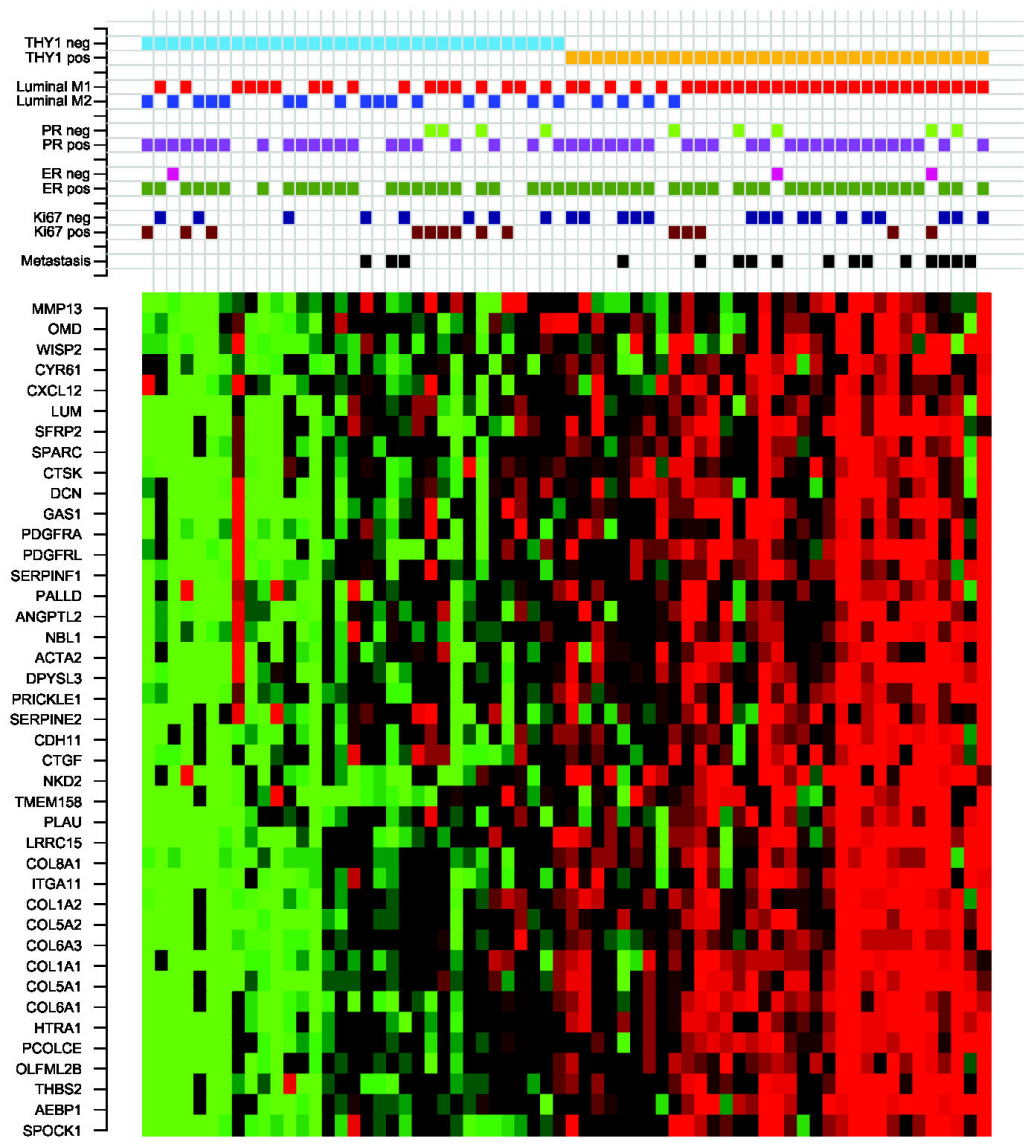
Heatmap of the genes in the module regulated by *THY1* in MBC. Red corresponds to up-regulation and green to down-regulation. The 66 MBC tumors are sorted according to their gene expression level of *THY1*.

**Figure 3:**
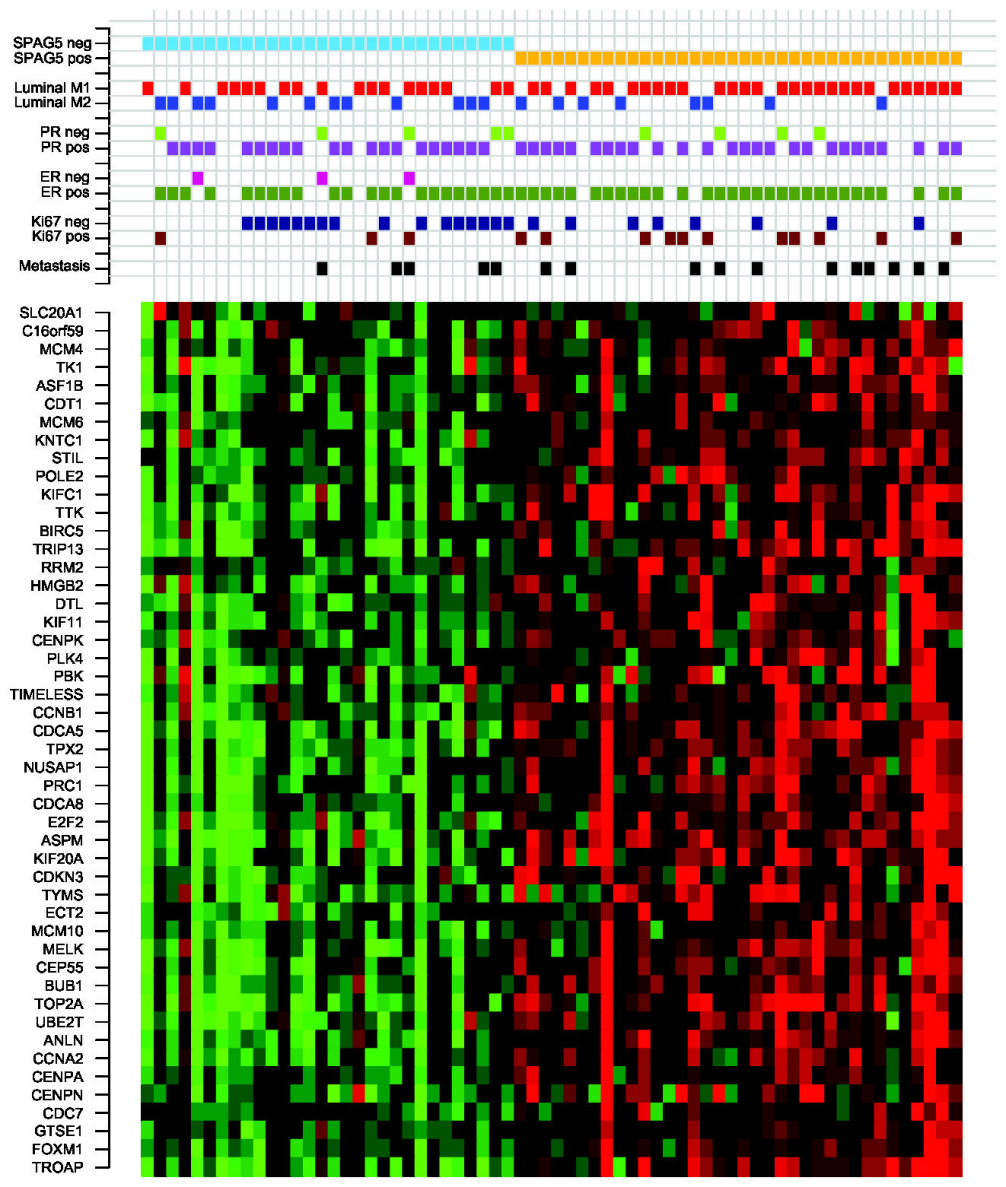
Heatmap of the genes in the module regulated by *SPAG5* in MBC. Red corresponds to up-regulation and green to down-regulation. The 66 MBC tumors are sorted according to their gene expression level of *SPAG5*.

**Figure 4:**
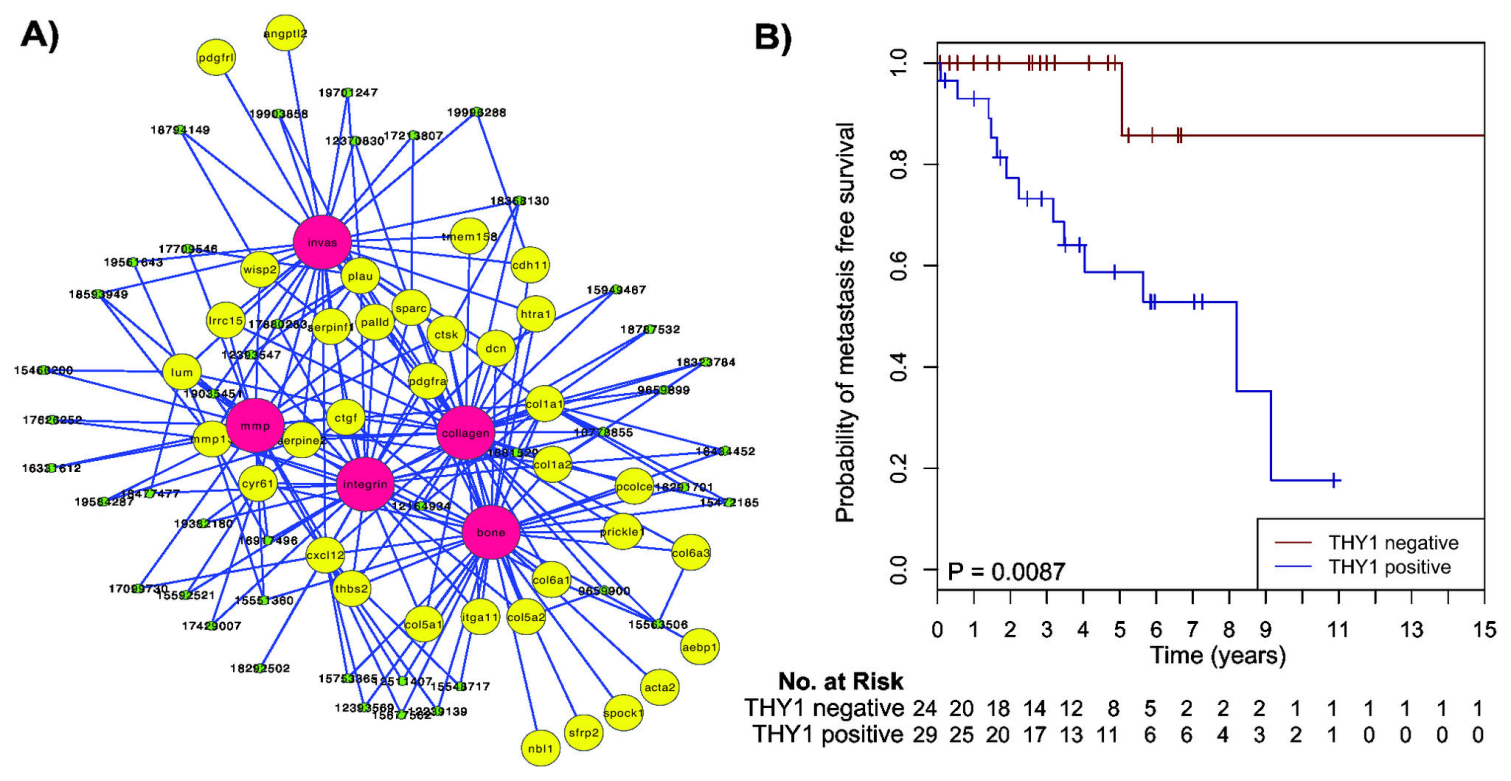
Graphic output of the LitVAn result for the MBC *THY1* module and corresponding Kaplan-Meier survival analysis. **A**) Significantly over-represented terms are represented as red circles and their association (graph edges) with multiple genes (yellow circles) from the module. The green dots represent the publication that significantly associates between the gene and the term, and the numbers in the green dots are the PubMed IDs for the respective publications. **B**) Distant metastasis free survival of the 66 MBC patients stratified by *THY1* gene expression. The numbers below the plots indicate the number of patients at risk in each group at the given time points.

**Figure 5:**
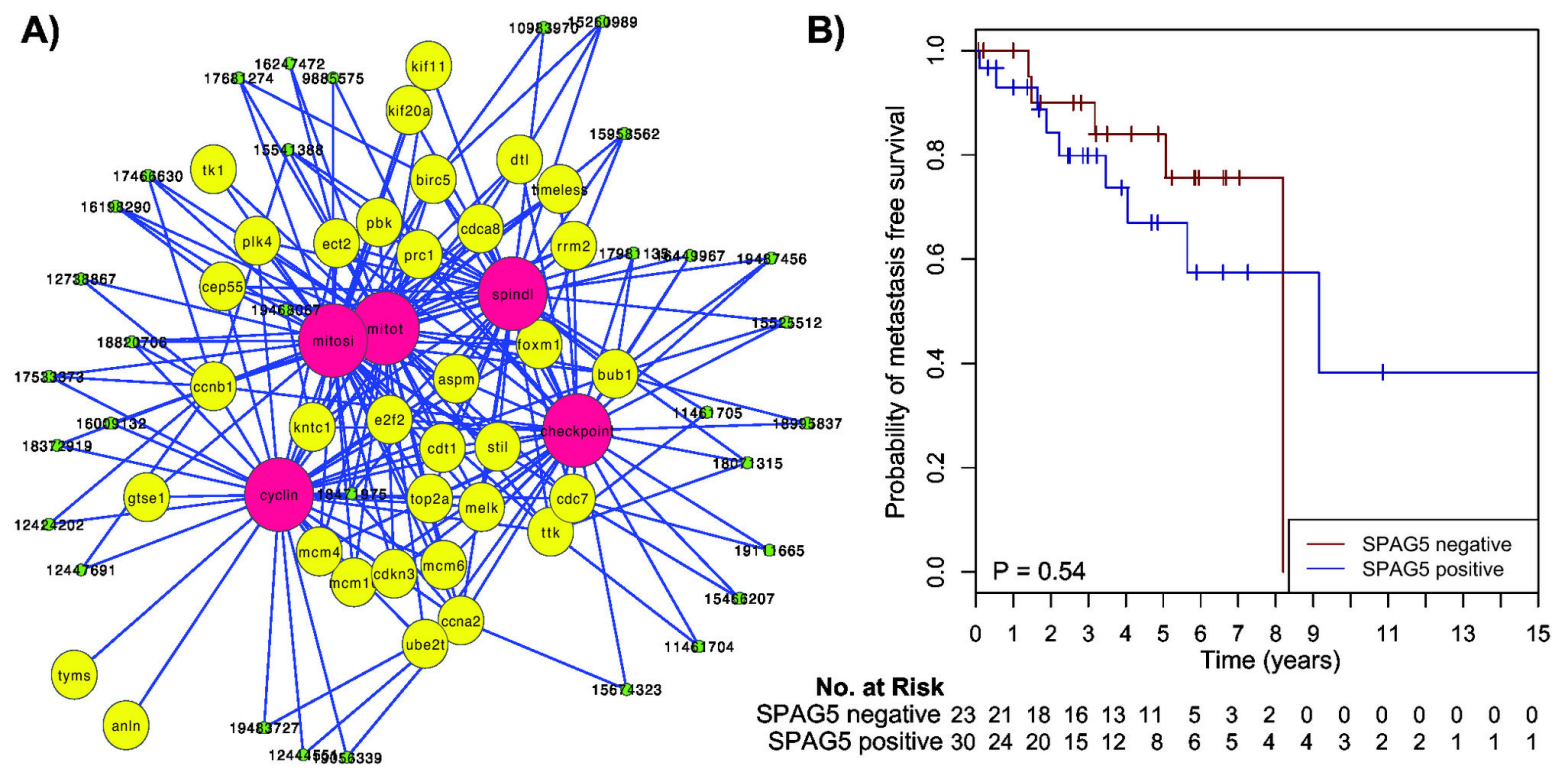
Graphic output of the LitVAn result for the MBC *SPAG5* module and corresponding Kaplan-Meier survival analysis. **A**) Significantly over-represented terms are represented as red circles and their association (graph edges) with multiple genes (yellow circles) from the module. The green dots represent the publication that significantly associates between the gene and the term, and the numbers in the green dots are the PubMed IDs for the respective publications. **B**) Distant metastasis free survival of the 66 MBC patients stratified by *SPAG5* gene expression. The numbers below the plots indicate the number of patients at risk in each group at the given time points.

Each potential driver is associated with gene modules (examples of two modules for MBC are shown in Figures 2-3) and the tumors are split into two groups for each candidate driver with a gene expression split value for that candidate driver. The two groups for each candidate driver display differential expression patterns of the genes in the modules, and these modules can help to explain the biological processes and pathways regulated by the candidate drivers. This information makes it possible to identify the most biologically and clinically interesting candidate drivers. LitVAn was used to perform text mining on the 97 MBC modules. A number of over-represented terms were thus found for MBC, including ‘cyclin’, ‘p53’, ‘tgf’, ‘invasion’ and ‘metastasis’ (Tables 2-3 and Figures 4-5). 

When gene set enrichments using gene ontology (GO) terms for the modules were studied to identify terms that were significantly enriched among MBCs, terms such as ‘cell differentiation’, ‘cell cycle’, ‘division’ and ‘signal transduction’ were found. Consequently, many of the candidate driver genes identified are known to be involved in processes associated with tumorigenesis, such as proliferation, motility, invasion, metastasis and cell differentiation. 

### THY1, a candidate prognostic invasion marker in MBC

We used the results from the LitVAn text mining and GO enrichment analysis for the selection of candidate driver genes of biological and clinical relevance for further validation. One of the most interesting modules was regulated at the first split by *THY1* (Thy-1 cell surface antigen, or *CD90*) and contained 41 genes; the text mining revealed over-represented terms including ‘collagen’, ‘invasion’, ‘mmp’, ‘integrin’, ‘angiogenesis’, and ‘tgf’ (Figures 2 and 4). The THY1 module was enriched for GO terms including cell adhesion and collagen. Taken together, these findings suggest that *THY1* plays a role in invasion and metastasis in MBC. The THY1 positive group in the present study was significantly associated with the previously described transcriptionally derived Luminal M1 group, and the THY1 negative group with the Luminal M2 group (Fisher’s exact test, P=0.0027). Moreover, the Luminal M1 group had a significantly higher module score for the invasion and metastasis module represented by *PLAU*, further supporting that *THY1* is involved in invasion and metastasis in MBC [2]. *THY1* maps to chromosome 11q22.3 and is a glycophosphatidyl-inositol (GPI)-anchored cell surface glycoprotein with a number of biological functions involving cell-cell and cell-matrix interactions and cell motility [28]. Furthermore, *THY1* is included as an up-regulated gene in the EMT core signature defined by Taube et al., and the module that it regulates contains seven of these up-regulated EMT genes. The genes up-regulated in the EMT core signature were also significantly up-regulated in the THY1 positive MBCs, while the down-regulated genes were not differentially expressed across the MBC tumors (Figure S1) [29].

The LitVAn text mining also revealed ‘tgf’ as an over-represented term in MBC and TGFβ signaling is known to play an important role in EMT [30], further supporting that the THY1 module is involved in EMT. Fredlund et al. identified an EMT-induced stroma module correlating with aggressive disease in the Basal-like subtype of FBC [31]. The THY1 module identified among the MBCs in the present study contained 50% of the genes in this stroma module and the THY1 positive MBC tumors displayed significantly higher activity for the stroma module (Figure S2). Furthermore, the THY1 negative tumors displayed low activity for the steroid response module, which is also in agreement with our earlier results, where the Luminal M1 tumors displayed low activity for the ER module [2].

EMT has been shown to be closely associated with the claudin-low subtype of breast cancer in earlier studies [29,32]. A claudin-low signature was therefore used to cluster the MBC tumors in the present study, but no correlation with the claudin-low subtype was found (data not shown) [33]. Next, the 66 MBC tumors included in our original transcriptional profiling study were split into two groups using the gene expression split value for *THY1*, resulting in two equally sized groups containing 33 patients each [2]. The 13 MBC tumors not included in the CONEXIC analysis in the present study displayed the same pattern as the 53 tumors included in the CONEXIC analysis (Figure 2). Importantly, *THY1* was found to be significantly associated with survival among MBC patients, with high expression of *THY1* predicting a significantly shorter time to DMFS (P=0.0087; Figure 4B). 

In order to validate the association between *THY1* expression and the genes in its module, an independent dataset with 37 MBC tumors was used [4]. The MBC tumors in the validation cohort were sorted according to increasing expression of *THY1*, whereupon a comparison of the heatmaps for the two datasets displayed highly similar patterns (Figure S3), adding support for *THY1* as a potential candidate driver in MBC. Moreover, the genes in the THY1 module were significantly correlated to *THY1* compared to all other genes in both datasets (Wilcoxon test, P=2.2e-16 and P=6.0e-11, respectively). 

### SPAG5, a potential driver of cell proliferation in MBC

Another biologically and clinically interesting module was regulated at the first split by *SPAG5* (sperm-associated antigen 5) and contained 48 genes (Figure 3). LitVAn found ‘cyclin’, ‘mitot’, ‘mitosis’, ‘spindl’, ‘checkpoint’, ‘g2’, ‘nocodazol’, ‘g1’, ‘p53’ and ‘cdk2’ to be overrepresented terms (Figure 5A). The SPAG5 module had enrichment of GO terms including cell cycle, cell division, M phase, mitosis, spindle and regulation of cell cycle. These results indicate that the SPAG5 module is involved in the mitotic checkpoint and progression of MBC, and that *SPAG5* is a potential driver of proliferation in MBC. *SPAG5* maps to chromosome 17q11.2 and encodes a protein, also called astrin, associated with the mitotic spindle machinery [34]. Fredlund et al. identified two modules involved in proliferation in breast cancer, the mitotic checkpoint module and the mitotic progression module [31], and the SPAG5 module identified herein among MBCs contained 70% of the mitotic checkpoint genes and 100% of the mitotic progression genes. The highly expressing *SPAG5* tumors displayed a significantly higher activity for the mitotic checkpoint and mitotic progression modules, while there were no differences for the other modules (Figure S4), further supporting that this module is involved in the proliferation of MBC. SPAG5 has been shown to be prognostic in ER positive, but not in ER negative breast cancer in women, while it has been shown to be predictive for chemotherapy response in the latter group [35]. Analysis of *SPAG5* in 1,379 FBCs in GOBO (Gene expression-based Outcome for Breast cancer Online) [36] confirmed that it was prognostic for distant disease free survival in ER positive, but not ER negative FBC (data not shown).

Next, the 66 MBC tumors included in our original transcriptional profiling study were split into two groups using the gene expression split value for *SPAG5*, resulting in one group containing 30 tumors with low expression of *SPAG5* and one group containing 36 tumors with high expression of *SPAG5*. The 13 MBC tumors not included in the CONEXIC analysis in the present study showed the same pattern as the 53 tumors included in the CONEXIC analysis. The SPAG5 groups were however not associated with the transcriptional Luminal M1 and M2 groups (Fisher’s exact test, P=0.18) [2]. Tumors in the SPAG5 high group had significantly increased levels of Ki67 compared to tumors in the SPAG5 low group (Wilcoxon test, P<0.05). However, when comparing survival between the groups, no significant difference could be seen (Figure 5B), which is in line with our earlier study where no prognostic value of Ki67 was seen in MBC [37].  

## Discussion

The development of high throughput and massively parallel techniques has moved forward rapidly and today many different methods for generating various different types of genome-scale data are available. As these techniques improve in resolution and scope, the complexity and heterogeneity of tumors is becoming increasingly evident. Cancer cells are equipped with multiple regulatory networks, enabling them to rapidly adapt to new contexts and to quickly grow and spread. This makes the task of eradicating tumor cells difficult and complex, and puts high demands on the development of therapy regimes. Mutations and chromosomal aberrations are central characteristics of tumors, and epithelial cancers harbor large numbers of such aberrations. It is therefore important to be able to distinguish between genetic changes that drive tumor progression and those that may be considered passenger events. An encouraging approach is to integrate different types of genome-scale data, which each shed light on different aspects of the underlying biology, to better understand which genes drive tumorigenesis, and the networks that they regulate. This may help us to better understand the complexity of tumors and move us closer to truly personalized cancer treatment.

The strategy employed by CONEXIC of identifying networks that the candidate drivers regulate has multiple advantages. Firstly, it gives a better understanding of the physiological context of the driver. Not all genes are targetable, thus targeting individual drivers may be impossible, but targeting suitable components of the networks might be a more amenable approach. A number of methods combining aCGH and GEX data for the detection of driver genes have been published to date [38-40], and they all define an important driver gene by simultaneous copy number aberration and differential expression. This assumption may however not always be valid for defining a cancer driver, as copy number alteration is only one of many means of altering the expression level of a gene. CONEXIC does not make this assumption [13], and may hence identify important driver genes that the other methods do not detect. Nonetheless, CONEXIC does not identify all potential cancer drivers, since it only detects drivers among amplified or deleted regions that pass the stringent statistical tests and will thus miss drivers arising from for example point mutations [13]. Recent attempts to identify mutations in driver genes in FBC have shown marked heterogeneity between individual tumors, as only 3-7 genes have been found to be mutated in more than 10% of FBCs [41,42]. The task of identifying candidate drivers in breast cancer is hence not easy. Thus, to identify all driver genes in MBC a much larger patient cohort would be required, as well as information regarding e.g. mutations and epigenetic changes. Nevertheless, our cohort constitutes the most comprehensive and well-annotated collection of MBC tumors, and we believe that the CONEXIC analysis has revealed several interesting and novel potential candidate drivers of importance for breast cancer progression and aggressiveness in men. Notwithstanding, we anticipate the outcome of the ongoing international effort aimed at further enhancing our knowledge of the underpinnings of this malignancy in a much larger cohort [8]. Furthermore, deep sequencing of large numbers of MBC tumors will be required to establish the involvement of driver genes implicated in MBC tumorigenesis by mutational events. 

In this study, CONEXIC was applied to 53 MBC tumors and 359 FBC tumors. CONEXIC identified 30 candidate drivers in the MBC tumors and 67 in the FBC tumors, of which only two candidate driver genes were in common (*TAF4* and *CD164*). Three additional candidate driver genes (*ARHGAP30*, *COG3* and *SPAG5*) in common with MBC were found when CONEXIC was applied separately to the intrinsic subtypes of FBC. This illustrates that male and female breast tumors are very different and is in line with previous results showing differences between MBC and FBC on the genomic and transcriptional levels [1-4]. We recently reported two new subgroups of MBC (Luminal M1 and M2, respectively) that differed from the intrinsic subtypes of FBC and as such appeared to occur only among males [2], further supporting these differences.

The MBC candidate drivers included two known cancer genes, *LHFP* and *ZNF217*. In addition, when CONEXIC was applied to the ‘MBC unique’ gene set, *MAP2K4* was detected as a candidate driver. *MAP2K4* was not included in the ‘MBC/FBC common’ dataset. *MAP2K4* is involved in the JNK pathway and loss of this gene results in defective apoptosis in response to stress [43]. Mutations in *MAP2K4*, which is a substrate for MAP3K1, have been seen as a rare event in Luminal FBCs, and pathway analysis showed that it produces similar pathway perturbations as *MAP3K1* mutations [44]. Furthermore, *MAP2K4* deletions were detected predominantly in ER positive tumors in a comprehensive study of 2,000 FBC tumors [45], suggesting that MBC tumors share some features with ER positive FBC. 

Many known drivers of breast cancer, including *GATA3*, *CCNE1*, *CDK4* and *GRB7*, as well as known drivers for other tumor types [26,27] were identified among the FBC candidate drivers. Ellis et al. also found *GATA3* to be a driver of FBC and they suggested that *GATA3* mutations might be a positive predictive marker for response to aromatase inhibitors [44]. In another study, Dutta et al. found *GATA3* to be a driver specifically for ER positive FBC. We identified *GATA3* as the top candidate driver among all FBC tumors and also for Luminal A and Luminal B FBC tumors. *GATA3* was however not identified among the candidate drivers in the MBCs in the present study, despite the majority of these tumors being ER positive. Taken together, these findings suggest that MBC tumors appear to share features with both ER positive and ER negative FBC tumors.

In all, the LitVAn study and GO enrichment analysis of the gene modules showed that many of the candidate drivers identified in male as well as female breast cancer are known to be involved in processes associated with tumorigenesis. 

*THY1* was one of the most biologically and clinically interesting candidate drivers for MBC, and it seems to be a driver of invasion related to EMT. Interestingly, the THY1 groups were highly correlated with the previously described Luminal M1 and M2 groups, despite being identified independently of these groups. This finding thus further validates that the transcriptional MBC groups represent two stable subgroups of MBC [2]. There was a significant difference in survival between the THY1 groups, where high expression of *THY1* correlated to poor prognosis. Interestingly, this phenotype also corresponds to the claudin-low subtype of triple-negative breast cancers, but MBC tumors are not classified as claudin-low. Furthermore, *THY1* expression levels were significantly correlated to the target genes in the module compared to all other genes, supporting a role for *THY1* in MBC. The module regulated by *THY1* was highly correlated to the stroma module described by Fredlund et al. in FBC, and high stroma module activity correlated with poor outcome in Basal-like FBC tumors [31]. The high expressing THY1 group of MBCs also displayed a significantly decreased activity for the steroid response module, a feature common to ER negative breast cancers, further indicating that these tumors behave more like ER negative than ER positive FBC, despite the majority of the MBCs being ER positive. This is in line with our earlier study where the Luminal M1 group, which correlates with the high expressing THY1 group, showed decreased activity for the ER module. However, the Luminal M2 group, while most resembling Luminal A FBCs, was also found to differ from the conventional intrinsic subgroups of FBC. Here, we show that the THY1 negative group, containing the majority of the Luminal M2 tumors, had low activity for the stroma module, which is one of the highest scoring modules for Luminal A tumors [2]. This further confirms that the heterogeneity observed among MBCs on the transcriptional and genomic levels is not readily captured with the instrinsic subtypes of FBC. 

Of interest, *THY1* has been shown to have contradictory functions in different tumor types, where it functions as a tumor suppressor in ovarian [46] and nasopharyngeal cancer [47], yet it promotes migration and metastasis in melanoma [48] and hepatocarcinomas [49]. 

Another potential driver of biological and clinical interest for MBC identified in the present study was *SPAG5*, the expression of which likely explains the differentially expressed genes involved in mitotic progression and mitotic checkpoint control, and illustrating that proliferation is an important feature of MBC. Proliferation is one of the most important determinants separating Luminal A from Luminal B tumors among ER positive FBC tumors, and patients with highly proliferative Luminal B tumors have a significantly inferior outcome compared to patients with Luminal A tumors [50]. Despite the majority of the MBC tumors being ER positive, they did not behave like ER positive FBC tumors, as no difference in survival between the two SPAG5 groups was detected although the SPAG5 module was linked to proliferation. Most probably the *SPAG5* negative tumors harbor other features than high proliferative that render them aggressive. A recent study showed that SPAG5 was prognostic in ER positive, but not in ER negative FBC, while it was predictive for response to chemotherapy in ER negative FBC [35]. Unfortunately, we were not able to stratify the SPAG5 groups on chemotherapy, as only 5 patients received chemotherapy. SPAG5 may be predictive for chemotherapy response also in MBC, in which case more MBC patients may benefit from chemotherapy. 

In the context of MBC this is a large collection of fresh frozen MBC tumors, but we need to keep in mind that the study is based on a small number of patients and further studies are needed to validate these findings. An international consortium for studying MBC has recently been formed, and clinical information as well as tumor material from a large number of MBC patients around the world is currently being collected with the aim to enhance our understanding of MBC [8].

In summary, the combination of copy number and gene expression data revealed vast differences in the landscapes of candidate drivers between male and female breast cancers. Furthermore, these results suggest that although the vast majority of MBCs are ER positive they share many features with ER negative FBC. Consequently, the pathobiology of MBC may be very distinct from that of FBC, and men diagnosed with breast cancer may therefore require different management and treatment strategies than women. Furthermore, THY1 is a promising prognostic invasion marker for MBC, and the chemotherapy treatment predictive value of SPAG5 in MBC warrants further investigation.

## Supporting Information

Figure S1 **The mean expression values of genes up-regulated or down-regulated by the EMT core signature in the THY1 groups.** The up-regulated genes from the EMT core signature were significantly up-regulated for the THY1 positive tumors, while the down-regulated genes were down-regulated for all MBC tumors.
(EPS)

Figure S2 **The module activity of the eight gene modules reflecting biologically relevant transcriptional programs found in FBC in the THY1 groups.** *THY1* positive tumors had significantly higher module activity for the stroma module and significantly lower activity for the steroid response module.
(EPS)

Figure S3 **Validation of the THY1 module in an external MBC dataset.** The heatmaps of the THY1 module genes revealed similar transcriptional profiles in our dataset (**A**) and the external validation dataset (**B**). Red corresponds to up-regulation and green to down-regulation. The genes are sorted in the same order and the MBC tumors are sorted according to increasing expression of *THY1*.
(EPS)

Figure S4 **The module activity of the eight gene modules reflecting biologically relevant transcriptional programs found in FBC in the SPAG5 groups.** *SPAG5* positive tumors had significantly higher module activity for the mitotic checkpoint and mitotic progression modules.
(EPS)

Table S1 **All MBC candidate drivers.**
(DOCX)

Table S2 **All FBC candidate drivers.**
(DOCX)
