# Supplementary material for: The Landscape of Candidate Driver Genes Differs between Male and Female Breast Cancer
Source: PLoS One. 2013 Oct 23;8(10):e78299. doi: 10.1371/journal.pone.0078299 (PMC3806766; doi:10.1371/journal.pone.0078299)
Supplement: Table S1 — All MBC candidate drivers. (DOCX) [file pone.0078299.s005.docx]

**Table S1.** All MBC candidate drivers.

| Candidate driver | CONEXIC score |
| --- | --- |
| BLCAP | 2,544 |
| ELAC2 | 1,960 |
| THY1 | 1,586 |
| LHFP | 1,309 |
| LAD1 | 1,209 |
| CYC1 | 1,202 |
| DDX51 | 1,180 |
| ARHGAP30 | 1,085 |
| CD164 | 1,016 |
| SPAG5 | 823 |
| POSTN | 815 |
| ELF1 | 802 |
| FYN | 715 |
| TAF4 | 693 |
| LAMA4 | 607 |
| TUBB6 | 454 |
| WDR46 | 446 |
| ZNF217 | 446 |
| POLE2 | 397 |
| COG3 | 316 |
| B4GALT3 | 299 |
| SRP68 | 279 |
| NEK8 | 254 |
| TRAPPC4 | 229 |
| IPO09 | 187 |
| PARP10 | 154 |
| PIGS | 148 |
| ZNF18 | 91 |
| HYOU1 | 14 |
| MCM5 | 12 |
