# Supplementary material for: The Landscape of Candidate Driver Genes Differs between Male and Female Breast Cancer
Source: PLoS One. 2013 Oct 23;8(10):e78299. doi: 10.1371/journal.pone.0078299 (PMC3806766; doi:10.1371/journal.pone.0078299)
Supplement: Table S2 — All FBC candidate drivers. (DOCX) [file pone.0078299.s006.docx]

**Table S2.** All FBC candidate drivers.

| Candidate driver | CONEXIC score |
| --- | --- |
| GATA3 | 15,464 |
| ARHGAP19 | 15,179 |
| TIMP2 | 10,917 |
| YIF1B | 10,200 |
| APOM | 9,563 |
| POLR2F | 9,346 |
| NCAPG2 | 8,994 |
| CD4 | 8,299 |
| DIAPH3 | 8,176 |
| AIF1 | 7,723 |
| KIFC1 | 6,012 |
| TCF4 | 5,901 |
| PRR7 | 5,767 |
| CSNK2B | 5,107 |
| NISCH | 4,932 |
| SAR1B | 4,749 |
| SOCS1 | 4,603 |
| ANKRD11 | 4,517 |
| THBS2 | 4,239 |
| LYZ | 4,207 |
| SOX10 | 4,148 |
| TAF4 | 3,779 |
| GATS | 3,672 |
| CCNE1 | 3,156 |
| CUL5 | 2,989 |
| GTSE1 | 2,889 |
| TST | 2,716 |
| MRPL9 | 2,677 |
| PRCC | 2,530 |
| EIF4G3 | 2,437 |
| CDK4 | 2,357 |
| TXNL4A | 2,149 |
| SPRY2 | 2,129 |
| ELL | 2,084 |
| NCF4 | 2,033 |
| RNASEL | 1,506 |
| PPP1R13L | 1,437 |
| KIAA0196 | 1,399 |
| CUTA | 1,355 |
| LMO4 | 1,247 |
| TAPBPL | 1,166 |
| NELF | 1,119 |
| FN3KRP | 1,101 |
| CCDC76 | 1,064 |
| DHX40 | 1,013 |
| WWC2 | 993 |
| GRB7 | 993 |
| COL4A3BP | 918 |
| CD164 | 864 |
| B4GALNT1 | 855 |
| SEP15 | 838 |
| MMP25 | 832 |
| MYO7A | 766 |
| YTHDC2 | 719 |
| NUFIP2 | 688 |
| HEXB | 685 |
| PSENEN | 670 |
| MAP7 | 541 |
| HSPA1L | 536 |
| NFIB | 490 |
| POP4 | 456 |
| PPAPDC1B | 385 |
| DCP2 | 383 |
| CDC16 | 339 |
| ATXN10 | 296 |
| VAMP1 | 182 |
| CDH1 | 182 |
